# Research on the Limit Values of Reclamation Based on Ecological Security: A Case Study of Tongzhou Bay in Rudong, Jiangsu Province

**DOI:** 10.3390/ijerph19148301

**Published:** 2022-07-07

**Authors:** Haifeng Zhang, Lin Zhao, Wen Du, Qing Liu, Yifei Zhao, Min Xu

**Affiliations:** 1 School of Marine Science and Engineering, Nanjing Normal University, Nanjing 210046, China; 13906031603@tio.org.cn (H.Z.); 09347@njnu.edu.cn (L.Z.); w76004@njnu.edu.cn (W.D.); yfzhao@njnu.edu.cn (Y.Z.); 2 Island Research Center, Ministry of Natural Resources (MNR), Fuzhou 350400, China

**Keywords:** limit values, coastal reclamation, ecological security, Rudong, Jiangsu

## Abstract

Due to the growing demand for land resources, many coastal reclamation projects have been implemented around the world in recent decades. Although coastal zone reclamation provides economic benefits, it produces a series of threats to coastal environments and ecosystems. Hence, the ecological costs and economic benefits of reclamation projects must be balanced. In this study, we select Tongzhou Bay, a key development area of the marine industry in the Jiangsu Province, as the research region to study the limits of reclamation control of the port operation area based on regional ecological security. First, we determine the standard limit values of the tidal catchment water line and the water dividing line, the change rate of the tidal flux ±5%, and water area of sandbars above the 0 m line based on key factors and evaluation indices for the ecological impact of reclamation in this region. Then, eight reclamation cases are investigated in Tongzhou Bay, which include the undeveloped natural state, development status, construction projects to be built, and possible construction scale based on the results of tidal current numerical simulation calculations. Although case 3 has impacts on Section 2 (DM2) tidal flux of less than 5% and on Section 1 (DM1) tidal flux of less than 10%, it causes a northward shift of the flood catchment water line in the middle of Yaosha. Finally, case 8 meets the requirements of the standard limit values of evaluation indicators, e.g., 1455 hectares of reclamation is the limit value to maintain the natural state of the Sanshahong channel and the stability of the tidal creek system and Yaosha. Therefore, the results suggest optimizing the structure and layout of breakwaters, controlling the restriction of reclamation, and further maintaining and protecting the ecological function of Tongzhou Bay.

## 1. Introduction

The coastal zone is an important interface of interaction between land and ocean and is also the zone with the most rapid and sensitive responses to global change and various dynamic effects of land and sea [1]. Meanwhile, coastal zones contain some of the most biologically diverse and productive ecosystems globally [2], which provide valuable ecosystem services, including protecting the shoreline from erosion, providing buffer zones and habitats for migratory birds, and increasing carbon storage and sequestration [3,4,5,6]. Therefore, the coastal zone is a key area of human society with relatively advanced socioeconomic development, and more than 40% of the population is concentrated within 100 km of a shoreline [7]. As a result, human activities, including industrial manufacturing, port development, and urban construction, have pervasive impacts on coastal ecosystems. Accelerating economic development and expanding urbanization in coastal areas over the last few decades can bring about land shortage problems for coastal zones [8]. Coastal reclamation is a promising strategy for creating and extending space for land resources, urban construction, and economic development globally [9]. In many coastal around the world, land reclamation is commonly practiced to accommodate economic growth [10,11,12,13]. Although coastal reclamation has brought rich economic benefits to local areas and promotes regional economic development, it has resulted in coastal pollution, habitat fragmentation, the loss of natural habitats, changes to the nearshore environment and further threats to ecosystem services [14,15,16,17]. Therefore, balancing the ecological costs and economic benefits of reclamation projects is necessary.

Tongzhou Bay is a key development area of the marine industry in Jiangsu Province. In recent years, the operation area of Tongzhou Bay has relied on the construction of a network of warehouse flood channels, which has led to development of a 200,000-ton entry channel and provides specific carrying space for a new container outlet of the Yangtze River. This area has a high potential for the development of agri-aquaculture, as well as the construction of the new deep-sea port along (e.g., Yaosha and Lengjiasha) deep tidal channels (e.g., Xiaomiaohong and Sanshahong) separated by large intertidal mudflats; this area is a typical silt tidal flat and coastal wetland habitat. At the same time, the harbor pool was reclaimed in Yaosha, and a channel and port wharf were built in the north. These reclamation activities have been shown to be linked to the decline of wetland ecosystems and endangered shorebird species. Considering the planning layout and the needs of gradual construction of the Tongzhou Bay operation area, ecological assessment should be carried out before and after the project construction and the subsequent phased development, and limits to reclamation control of the port operation area should be proposed based on regional ecological security. Therefore, the specific aims of the study were to (1) determine the key factors and evaluation indices of the ecological impact of reclamation in this region; (2) analyze the trend relationship between the evaluation index and reclamation plan and determine the assessment index and standard limit values; and (3) calculate the reclamation control limits for the stability of the ecological function of Yaosha based on regional marine ecological security.

## 2. Materials and Methods

### 2.1. Study Area

**Figure 1:**
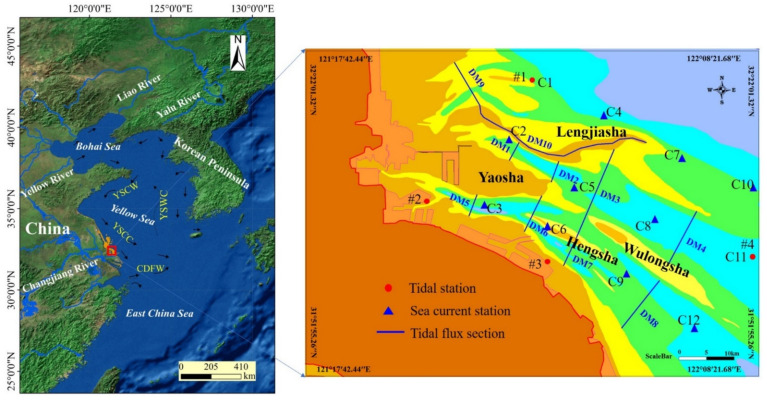
The map of study area.

Tongzhou Bay is located in the eastern Tongzhou District, Nantong city, Jiangsu Province, and the northern Yangtze River estuary (Figure 1). There are abundant tidal flat resources in this area, and it has developed large-scale mariculture and some port industries, including Lusi Port and Dongzao Port, which will be the key areas of marine economic development in Jiangsu Province in the future. Tongzhou Bay is located in the northern subtropical monsoon climate zone, with four distinct seasons, mild temperatures, and abundant rain. The annual average temperature is 16.1 °C, and the annual average precipitation is 1045 mm. Northerly winds prevail in winter, and southeasterly winds prevail in summer. The annual average wind speed is 6.8 m/s, and the annual average relative humidity is 75.8%.

The coastal area of Tongzhou Bay is located in the southern part of the Yellow Sea, and the tidal current is a regular half-day tidal current. When the tidal wave propagates from the East China Sea to the Yellow Sea, it maintains the characteristics of a forward wave along the southern coast of Jiangsu Province, and the Xiaomiaohong channel is mainly controlled by the East China Sea forward wave. The inshore tidal current is affected by the topography of the channel and sandbanks. The main flow direction of tidal currents in the deep channel is consistent with the strike of the channel and shows obvious characteristics of reciprocating flow. The tidal current in the shoal area of sandbanks mainly takes the form of the flood tide overflowing shoals and the ebb tide returning to the channel. The elevation of the study area ranges from −22.21 m to 8.06 m (mean sea level datum), and the annual mean low water spring tide is −1.93 m. The submarine topography consists of alternating sand ridges and channels, and the geomorphology shows complex changes, e.g., the Yaosha, Lengjiasha, Xiaomiaohong channel, Sanshahong channel, and Lengjiasha are distributed successively.

### 2.2. Data

In April 2019, we set up 12 temporary tide level stations (C1~C12, as shown in Figure 1) in the waters of Tongzhou Bay to conduct simultaneous observation of large, medium and neaps, and obtain measured tide level, tidal current, sediment, and other data.

### 2.3. Methods

#### 2.3.1. Numerical Model

First, a tidal wave mathematical model of the East China Sea is established. The model region is 117~131° E, 24~41° N, and the calculation range includes the Bohai Sea, the Yellow Sea, and the East China Sea. Based on large-scale tidal wave simulation, a tidal current mathematical model of the whole South Yellow Sea is developed. Based on the boundary conditions provided by the mesoscale model, a mathematical model of the local tidal current in the study area is established, and the model ranges from Qinghai Cape in Shandong Province to near the Taizhou Sea in Zhejiang Province.

**Figure 2:**
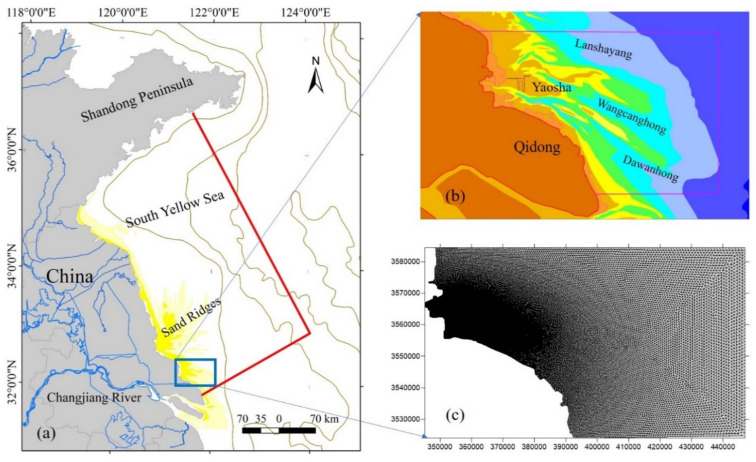
Computational domains of Jiangsu regional model (**a**), and Tongzhou Bay scope (**b**) and model (**c**).

According to the topography and tidal current characteristics of the sea in the study area, an area with a length of 100 km and a width of 50 km is selected as the calculation range of the mathematical model. In the calculation area of the model, the western boundary is the current shoreline, which is a closed boundary. The eastern, northern, and southern boundaries are open sea boundaries (Figure 2). A two-dimensional numerical model of the Tongzhou Bay sea area is established based on MIKE 21 software, and unstructured grids are used, with a total of 911,267 grids. The grid is of variable step size, and the grid scale is between 1 and 2000 m. The grid is dense in the area close to land and sparse in the open sea.

#### 2.3.2. Model Validation

**Figure 3:**
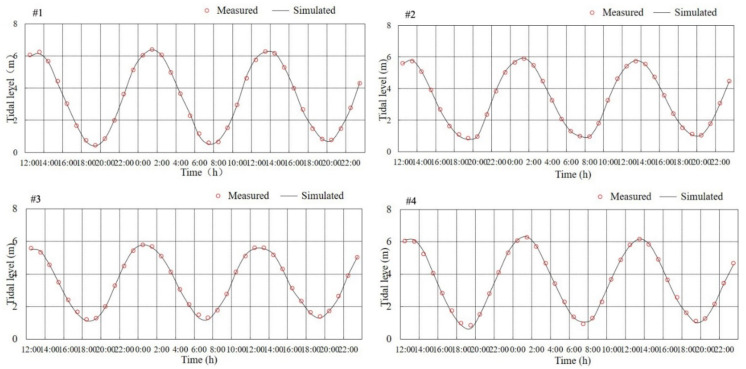
Validation map of spring tide level in April 2019 (local theoretical lowest tide level).

The mathematical model is verified based on the tidal level data from 4 tide stations and the measured hydrological and sediment data along 12 vertical lines collected in April 2019 in the study area (Figure 1). Figure 3 shows that the results calculated by the model for the spring tidal level are consistent with the measured values; the phase deviation of high and low tides is within 0.5 h, and the error of the tide value is within 10 cm. The verification results for the spring flow velocity and flow direction indicate that the deviation between the calculated value of maximum flow velocity and average flow velocity and the measured value is within 5%, and the deviation in flow direction is within 10° (Figure S1). In addition, the validation results for the magnitude and fluctuation of sediment concentrations calculated by the model are basically consistent with the measured data (Figure S2). Consequently, the verification results confirm that the established mathematical model can well reproduce the distribution characteristics of the marine dynamic field and the sediment field in the study area.

## 3. Results and Discussion

### 3.1. Selection of the Ecological Evaluation Indices and Limit Values of Reclamation in Tongzhou Bay

Coastal wetlands are the main ecological feature of Tongzhou Bay, and their ecological function is to provide the habitat foraging grounds for benthic organisms and birds. Yaosha and Lengjiasha are the main coastal wetlands in the study area, and the stability of sandbanks is the material and environmental basis for maintaining their ecological functions. The movement of the Sanshahong and Xiaomiaohong channels directly affects the morphological changes in Yaosha and Lengjiasha and then leads to morphological and area changes in the coastal wetlands, which results in the reduction or even partial loss of wetland ecological functions. Therefore, maintaining the stability of the water channel is the key to maintaining the stability of regional tidal flats and ecological security. The reclamation works in Tongzhou Bay are mainly concentrated on the Yaosha sandbank; this work will directly occupy part of the Yaosha coastal wetland and affect the surrounding hydrodynamics and the scouring and silting environment, resulting in the degradation of the Yaosha coastal wetland. Therefore, maintaining the ecological function of the Yaosha coastal wetland is an important basis of ecological protection in this region. Consequently, the ecological protection objectives for the construction of the Tongzhou Bay operation area are determined to be the stability of the sandbank and channel pattern and the stability of the ecological function of the Yaosha wetland.

The reciprocating flow characteristics of the Xiaomiaohong channel, Sanshahong channel, Dawanhong channel, and Wangcanghong channel are obvious, and the main flow directions of the tide in the deep channels are consistent with the directions of the channels. The tidal currents on the Yaosha and Lengjiasha shoals mainly involve the flood tide moving over sandbanks and the ebb tide returning to the water channel. Therefore, the tidal dynamic strength of the water channel is the decisive factor to maintain the stability of the channel. According to the technical guidelines for environmental impact assessment of bay reclamation planning (GB/T 29726-2013), the factors related to tidal current dynamic intensity, including tidal current velocity, flow direction, and tidal flux, can be selected as ecological assessment indicators. The standard limit value of the tidal flux change rate is ±5% based on the limit value requirements of tidal current dynamic stability discrimination.

The ebb tide dividing line and flood tide catchment line not only represent the dynamic environmental characteristics of the sandbank, but also affect the scour and silting evolution of the Yaosha area, which directly affects the ebb tide flow returning to the channel and the tidal dynamic strength in the Sanshahong and Xiaomiaohong channels. Therefore, the Yaosha catchment line and dividing line should also be used as ecological evaluation indices. The relatively stable positions of the catchment line and dividing line are the standard limit values of stability discrimination.

In addition, the sandbar area is periodically submerged by water associated with rising and falling tides, maintaining an exchange of matter and energy with the surrounding sea. Thus, the water area and water time above the 0 m line of Yaosha were selected as ecological evaluation indices and limit values.

In general, the rate of change in the tidal flux ±5%, the relatively stable positions of the catchment line and dividing line, and the water area and water time above the 0 m line of Yaosha can be used to determine the limit values of reclamation (Table 1).

### 3.2. Reclamation Project Case and its Environmental Impact

**Figure 4:**
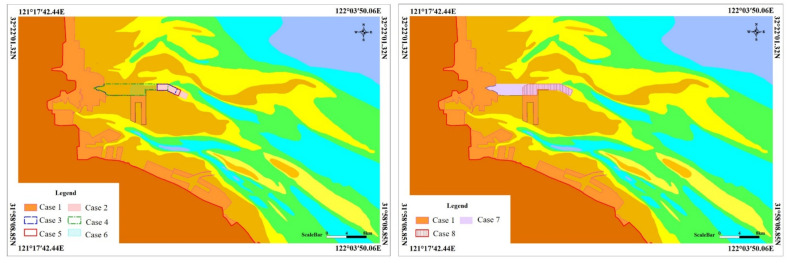
Reclamation project case of study area.

To study the control limit values of reclamation in the engineering area of Tongzhou Bay, tidal current numerical simulation calculations were carried out for several reclamation schemes in Yaosha, including the undeveloped natural state, development status, and possible construction scale according to the time sequence of port engineering construction in the study area. At the same time, the calculated results for the tidal flux, the water area and water time above the 0 m line, and the catchment line and dividing line of Yaosha were extracted, and the influence of reclamation schemes on tidal current field was analyzed and compared (Figure 4, Table 2).

After the implementation of the reclamation projects, the engineering area prevents water movement and deflects flow during flood and ebb tides. The construction of the dike cuts off part of a tidal creek, which affects the flow path of the local channel flow. However, the influence of engineering construction on the convective state is associated with the local adjustment of the flow state in the Sanshahong tidal water and has little influence on the confluence and return channel of the Sanshahong and Xiaomiaohong tidal grooves. Among them, the tidal state on the northern side of the middle part of Yaosha has certain deflections compared with the natural state in cases 2, 3, and 4 due to the effects of water resistance and flow diversion. In addition, the influence of the tidal state is mainly concentrated at the northern root of Yaosha in cases 5 and 6. Compared with the natural state, cases 7 and 8 change the flow patterns in the middle and tail sections of the Sanshahong channel and on the northern side of Yaosha (Figure S3).

**Figure 5:**
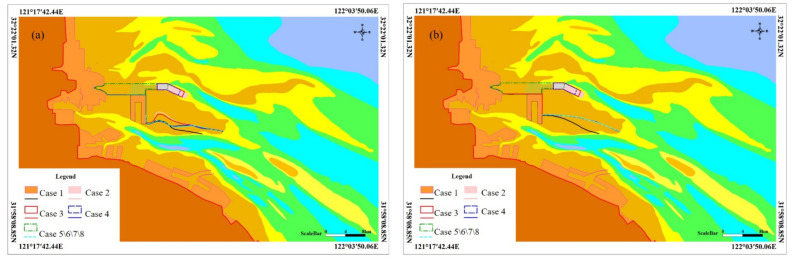
The change of catchment line (**a**) and dividing line (**b**).

The tidal current on the Yaosha shoal experiences flood tides overflowing sandbanks and ebb tides returning to the channel under natural conditions. At the end of a high tide, the tidal currents on both the southern northern sides of Yaosha converge with the rising tide level and form an obvious catchment line. At the beginning of an ebb tide, the water on the Yaosha surface flows downward, and from the higher topography of the Yaosha area, water flows into the Sanshahong channel and Xiaomiaohong channel along the southern slope and the northern slope of Yaosha, respectively. The dividing line where the tidal current turns to either side is the ebb tide dividing line. In the natural state, the water dividing line and the catchment line on Yaosha are distributed on the tidal flat surface in southern Yaosha because Yaosha topography is characteristically higher in the south and lower in the north (Figure 5).

In case 1, the water dividing line and catchment line on the Yaosha root move obviously northward with the direction of the engineering layout, which is mainly due to the water blocking effect of connecting the land passageway in the engineering area. After the implementation of case 2, the middle catchment line of Yaosha still moves northward, while the tail catchment line of Yaosha is basically consistent with that in the natural state. In addition, there is no obvious change in the water dividing line in Yaosha. After the implementation of case 3, the catchment line in the middle of Yaosha still moves northward. However, due to the decrease in the reclamation area compared with case 2, the northward migration of the catchment line decreases to a certain extent, and the distribution pattern of the catchment line at the tail of Yaosha is basically consistent with that in the natural state. There is no obvious change in the dividing line in Yaosha. The implementation of case 4 has a certain influence on the flow pattern only on the northeastern side of the front edge of the northern slope of the Yaosha engineering area but has a relatively small influence on the overplain flow pattern in the middle and tail of the northern slope of Yaosha, and the catchment line and dividing line have no obvious changes. The implementation of case 5 occupies the nearshore tidal flat, which has a certain influence on the flow regime in the northern part of the reclamation area, but the influence is mainly concentrated in the nearshore shoal in the northern part of the root of Yaosha and has little influence on the morphological distribution of the flood catchment line and ebb tide water dividing line in Yaosha. Case 6 reclamation mainly optimizes the layout of the reclamation area, and the flow regime changes caused by reclamation focus mostly on the nearshore shoal to the north of the root of Yaosha; these changes have little influence on the morphological distribution of the catchment line and dividing line in Yaosha.

Based on the calculated results for cases 2–6, cases 4, 5, and 6 have no obvious influence on the catchment line, and the amplitude of tidal flux on a typical section of the waterway is less than 5%. Therefore, the project cases in the east and west are superimposed to calculate the dynamic environment after superimposition. According to the calculated results, there is no obvious difference between cases 7, 8, and 1 in the flood catchment line and the ebb tide dividing line in Yaosha. Consequently, there is little change in the different cases of the ebb and flow dividing lines in Yaosha. The flood tide catchment line in Yaosha in case 2 and case 3 deviates northward to a certain extent compared with the natural state.

The tidal flux in the channel is an important factor reflecting the dynamic change in the deep channel. Reclamation occupies the Yaosha area and causes diversion and water resistance, which has a certain effect on the tidal flux in the channel. To evaluate the influence of various reclamation schemes on tidal flux, several sections were selected at different water depths in the Sanshahong and Xiaomiaohong channels to calculate the sectional tidal flux (Figure 1).

The statistical results (Table S1) show that compared with the current conditions, the variation range of the DM1 section in case 2 is greater than 10%, and those of the other sections are less than 5%. In case 3, the variation range of the DM1 section is less than 10%, and those of the remaining sections are less than 5%. In case 4, the variation range of the DM1 section is less than 10%, and those of the remaining sections are less than 5%. In case 5, the variation range of the DM1 section is greater than 10%, and those of the remaining sections are less than 5%. In case 6, the variation range of the DM1 section is less than 10%, and those of the remaining sections are less than 5%. In case 7, the DM1 section variation amplitude is greater than 10%, and those of the remaining sections are less than 5%. In case 8, the variation range of the DM1 section is less than 10%, and those of the remaining sections are less than 5%. Consequently, the tidal flux of each scheme has a different degree of change, and the variation range of tidal flux at the end of the channel is greater than that at the open sea. In addition, the variation range of the tidal flux in the section of the Sanshahong channel is greater than that of the Xiaomiaohong channel.

**Figure 6:**
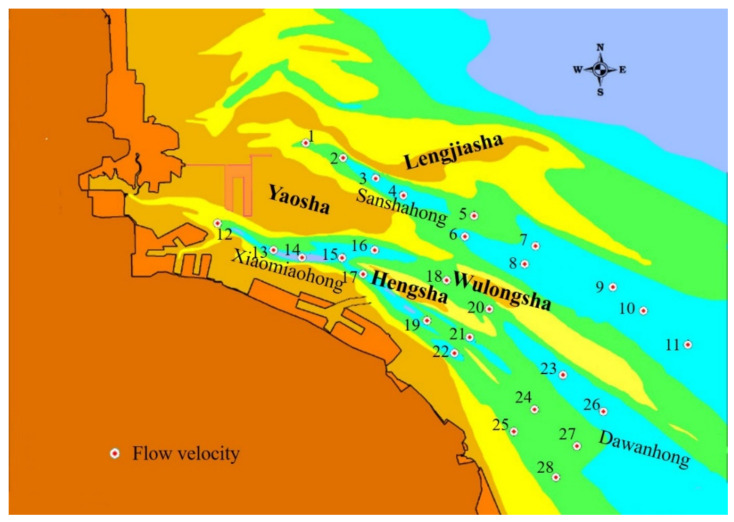
Location map of velocity in water channel.

Tidal current dynamics are an important factor affecting the stability of the water channel. To evaluate the influence of various reclamation schemes on the velocity in the water channel, several feature points are selected along the Sanshahong and Xiaomiaohong channels, among which feature points #2, #4, #5, #6, #9, #13, #16, #17, #18, #19, #23, #24, and #25 are located in each channel section of the deep channel (Figure 6).

The calculated results show that the velocity variation for each scheme is mainly concentrated at the ends of the Sanshahong channel and Xiaomiaohong channel. In all schemes, the influence is mainly concentrated on feature points #1 and #2 near the project. However, the flow direction changes are mainly concentrated in the shallow area with a depth of −10 m in the Sanshahong channel around the project (Table S2).

#### Impacts of Reclamation on the Change in Water Area and Water Time in Yaosha

According to the 2019 topographic mapping data, the area above the 0 m isobath in Yaosha is approximately 15,109.41 hectares. All reclamation schemes occupy tidal flats above the 0 m isobath line to varying degrees. The larger the scale of reclamation, the more the 0 m isobath tidal flat of Yaosha decreases. Except for case 5 and case 7, other schemes result in a smaller proportional decrease in the water area of Yaosha (Table 3).

The model is used to calculate the variations in water time in the area of Yaosha after the implementation of various reclamation schemes (Table 3). The model results indicate that with the implementation of each reclamation scheme, the time with water above the Yaosha ridge line is more than 5 h in a tidal cycle. Compared with other cases, case 2 and case 7 have certain reductions in water time, but the reductions are small. Consequently, each reclamation scheme has little influence on the water time of Yaosha in this study.

### 3.3. Determination of Reclamation Limit Value Scheme

According to the evaluation results (Table 4), case 4 will not cause the spatial movement of the flood catchment line and ebb and flow dividing line in Yaosha, the amplitudes of the DM2 ebb and flow fluxes of the control section are less than 5%, and the amplitudes of the DM1 ebb and flow fluxes of the reference section at the tail of the Sanshahong channel are less than 10%, which are all acceptable reclamation schemes. Although case 3 has an impact on DM2 tidal flux of less than 5% and a DM1 tidal flux of less than 10%, it causes a northward shift in the flood catchment line in the middle of Yaosha. Therefore, case 4 is suitable for the development plan scheme of the Tongzhou Bay operation area.

Case 5 and case 6 do not cause a shift in the spatial position of the flood catchment line and ebb and flow dividing line in Yaosha, the amplitudes of the two schemes on the flood and ebb flux of the DM1 section of Sanshahong are less than 10%, and the amplitudes of the flood and ebb fluxes of the DM2 section are less than 5%. Therefore, cases 5 and 6 are acceptable options for separate enclosure development in the future.

After the implementation of case 7, the variation range of the DM1 section is greater than 10% and that of the DM2 section is less than 5%. In case 8, the variation range of the DM1 section is less than 10% and that of the DM2 section is less than 5%. Therefore, case 8 still meets the requirements of the standard limit values of evaluation indicators, e.g., 1455 hectares of reclamation area is the reclamation limit value to maintain the natural state of the Sanshahong channel, the stability of the tidal creek system, and the stability of Yaosha.

In addition, except for case 2 and case 7, which cause small decreases in the water time of Yaosha, the other schemes basically have no effect on the current water time of Yaosha. Therefore, case 8 can maintain the ecological function of the Yaosha wetland after implementation.

### 3.4. Check Limit Value of Reclamation Based on the Stable State of the Topographic Deposition-Erosion Balance

Case 8 is the reclamation limit value scheme of the Tongzhou Bay operating area determined in this study, e.g., increasing the reclamation area by 1455 hectares in Yaosha. After the implementation of the project, the hydrodynamic environment of the study area will be affected, which will lead to topographic adjustment and changes in scouring and silting in the study area; the area will finally reach a new dynamic balance in a period of time. To verify whether there is any change in the limit values of reclamation on the basis of the new balance of scouring and silting in the study area after the implementation of case 8, numerical simulation calculations of topography scouring and silting are carried out, and each limit value factor of reclamation is checked.

**Figure 7:**
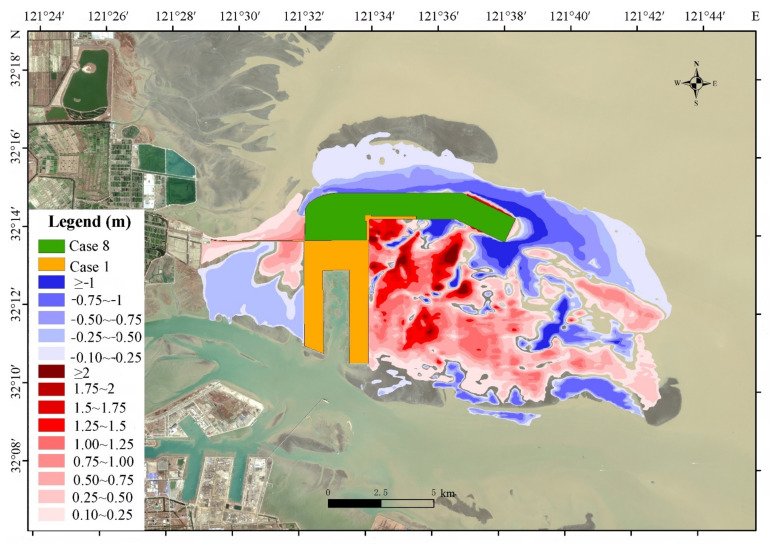
Topographic changes of model results in Yaosha.

The model results show that after the implementation of case 8 (Figure 7), the topographic changes in Yaosha are mainly dominated by siltation; the siltation of Yaosha near the offshore side is approximately 0.10–0.75 m, the siltation is approximately 0.10–2.0 m in the middle, and the siltation in the southern weak current area is the largest at approximately 1.0–2.0 m. The eastern and northern parts of the project area experience increase in hydrodynamic force due to the diversion of the reclamation, which results in erosion of the topography. The erosion depth at the front of the project area is greater than 1.0 m, while the erosion depth on the northern side is approximately 0.1–0.75 m.

Based on the stable deposition–erosion balance in the topography, tidal current and sediment mathematical simulation results show that case 8 causes no significant changes in the ebb and flow dividing line and catchment line, and the variation rate of the section flow is controlled within 10% (Figure S3).

In case 8, the tidal flux of each section is not obvious in the stable state of scouring and silting balance. The maximum variation rates of the DM1 and DM2 tidal fluxes in the control section are −4.66% and −3.06%, respectively, which are lower than the tidal flux limit requirements of the reclamation section (Table S3).

There is no significant difference in the variation in flow velocity at the feature points, and the variation amplitude of most feature points is less than 10%, except that the variation amplitude of feature point #1 is greater than 10% (Table S4).

Therefore, case 8 still meets the limits of reclamation indices after the evolution of scouring and silting, which is the limit value of reclamation to maintain the natural evolution trend of the Sanshahong channel, the stability of sandbanks and the water channel system, and the stability of the ecological function of Yaosha.

## 4. Conclusions

In this study, we select Tongzhou Bay, a key development area of the marine industry in Jiangsu Province, as the research region to study the limits of reclamation control of the port operation area based on regional ecological security. The results as follows:

(1) The stability of sandbanks is the decisive factor for maintaining the ecological function of the Yaosha wetland in Tongzhou Bay. According to the factors affecting the stability of sandbanks, the change rate of tidal flux ±5%, the relatively stable position of the diversion line and confluence line, and the water area and water time above the 0 m line in Yaosha are used to determine the limit values of reclamation.
(2) Eight reclamation schemes for Yaosha in Tongzhou Bay are selected, which mainly include the undeveloped natural state, development status, and possible construction scale according to the time sequence of port engineering construction. After the implementation of the reclamation projects, the engineering area prevents water influx and deflects flow during flood and ebb tides. The construction of the dike cuts off part of a tidal creek, which affects the flow path of the local channel flow. The ebb and flow dividing line shows little change in different schemes for Yaosha. The tidal flux of each scheme has a different degree of change, and the variation range of tidal flux at the end of the channel is greater than that at the open sea. Except for case 5 and case 7, the other schemes result in small proportional decreases in the water area of Yaosha, whereas the water time of Yaosha has little influence in this study.

## Figures and Tables

**Table 1:** Ecological evaluation indexes and standard limit values of reclamation.

Number	Indicator	Concepts and Types	Calculation Method	Limit Value
1	Catchment line and dividing line	The boundary line between the flood tide confluence and ebb tide divergence of sandbanks.	The identification of tidal current field at the end of high tide and the beginning of low tide is calculated via numerical simulation of the tidal current.	The catchment line and dividing line should be relatively stable compared with the current status.
2	Change in tide flux	The rate of change in the tidal flux at the time of maximum flood and maximum ebb along typical sections of the water channel.	Identification of tidal flux variability at 10 m depth sections in deep water channels before and after the project.	Control section at Sanshahong channel entrance: 5%; deep water channel tail reference section: ≤10%
3	Water area	The largest area submerged by ebb and flood tide water above 0 m on sandbars.	Identification of tidal level and water depth above the 0 m line of Yaosha.	-
4	Water time	The inundation time of tidal water in the area of sand ridge lines on sandbanks.	The tidal level and topographic recognition of ridge lines in Yaosha.	-

**Table 2:** Reclamation project cases.

Number	Project Case	Description of The Project Case
1	Case 1	Current situation of reclamation in Tongzhou Bay operation area
2	Case 2	Reclamation of approximately 790 hectares in the NE part of the current project area
3	Case 3	Reclamation of approximately 690 hectares in the NE part of the current project area
4	Case 4	Reclamation of approximately 585 hectares in the NE part of the current project area
5	Case 5	Reclamation of approximately 2470 hectares in the N part of the current project area
6	Case 6	Reclamation of approximately 1135 hectares in the N part of the current project area
7	Case 7	Case 4 + 5, reclamation of approximately 2790 hectares
8	Case 8	Case 4 + 6, reclamation of approximately 1455 hectares

**Table 3:** The changes of the water area and water time of Yaosha in different reclamation case.

Project Case	Overlap Area with Yaosha 0 m Line (ha)	Water Area (ha)	Change Rate	Water Time in a Tidal Cycle
Case 1	1217.32	13,892.09	/	5 h 40 min
Case 2	1299.79	13,809.61	−0.59%	5 h 35 min
Case 3	1299.79	13,809.62	−0.59%	5 h 40 min
Case 4	1299.93	13,809.48	−0.59%	5 h 40 min
Case 5	2435.13	12,674.28	−8.77%	5 h 25 min
Case 6	1677.49	13,431.92	−3.31%	5 h 40 min
Case 7	2485.91	12,623.5	−9.13%	5 h 25 min
Case 8	1710.99	13,398.42	−3.55%	5 h 40 min

**Table 4:** Comparison of different reclamation project cases.

Project Case	Yaosha Stability		Water Channel Stability										Change Rate of Water Area	Water Line
	Catchment Line	Dividing Line	DM1					DM2						
			Control the Maximum Variation Range of Section Flow	2# Control Point Maximum Flow Velocity Change		2# Control Point Maximum Flow Direction Change		Control the Maximum Variation Range of Section Flow	4# Control Point Maximum Flow Velocity Change		4# Control Point Maximum Flow Direction Change			
				Maximum Flood	Maximum Ebb	Maximum Flood	Maximum Ebb		Maximum Flood	Maximum Ebb	Maximum Flood	Maximum Ebb		
Case 2	The catchment line in the middle of Yaosha moved northward, and the distribution pattern of the tail catchment line was basically consistent with the natural state	No change	−11.58%	1.40%	−7.68%	1.35%	1.62%	−0.38%	−0.38%	−3.23%	−0.05%	−0.26%	−0.59%	5 h 35 min
Case 3	The catchment line in the middle of Yaosha moved northward, and the distribution pattern of the tail catchment line was basically consistent with the natural state	No change	−8.26%	−0.95%	−6.58%	1.11%	0.79%	−0.50%	−0.50%	−2.63%	−0.02%	−0.12%	−0.59%	5 h 40 min
Case 4	No change	−6.13%	−1.59%	−2.03%	0.91%	0.43%	−2.05%	−0.97%	−1.98%	0.02%	−0.05%	-	−0.59%	5 h 40 min
Case 5	No change	−7.37%	−4.17%	−6.06%	0.68%	1.42%	−2.52%	−2.49%	−2.45%	0.09%	0.02%	-	−8.77%	5 h 25 min
Case 6	No change	−6.36%	−3.06%	−5.85%	0.57%	1.07%	−2.05%	−1.91%	−2.09%	0.07%	0.02%	-	−3.31%	5 h 40 min
Case 7	No change	−11.20%	−3.09%	−7.58%	1.09%	0.81%	−3.09%	−1.64%	−3.23%	0.04%	−0.05%	-	−9.13%	5 h 25 min
Case 8	No change	−9.36%	−3.03%	−7.46%	1.09%	0.81%	−3.06%	−1.64%	−3.20%	0.04%	−0.05%	-	−3.55%	5 h 40 min

## Data Availability

Data supporting reported results can be presented by H.Z. on request.

## Supplementary Materials

The following supporting information can be downloaded at: https://www.mdpi.com/article/10.3390/ijerph19148301/s1, Figure S1: Validation of spring flow velocity and direction in April 2019; Figure S2: Validation of sediment concentration in water in April 2019; Figure S3: Influence of reclamation cases on the hydrodynamic environment; Table S1: Variation range of tidal flux at each section of the water channel; Table S2: The variation of flood tide velocity in each case; Table S3: Tidal flux variation of each section under stable topography with fixed bed and equilibrium in case 8%; Table S4: Characteristic point changes of flow velocity.
