# Supplementary material for: Research on the Limit Values of Reclamation Based on Ecological Security: A Case Study of Tongzhou Bay in Rudong, Jiangsu Province
Source: Int J Environ Res Public Health. 2022 Jul 7;19(14):8301. doi: 10.3390/ijerph19148301 (PMC9324534; doi:10.3390/ijerph19148301)
Supplement: Supplementary file 1 [file ijerph-19-08301-s001.zip › ijerph-1695369-SI.pdf]

Research on the limit values of reclamation based on ecological security: A case study of Tongzhou Bay in Rudong, Jiangsu Province

Haifeng Zhang [1,2], Lin Zhao [1], Wen Du [1], Qing Liu [1*], Yifei Zhao[1] and Min Xu[1*]
(1. School of Marine Science and Engineering, Nanjing Normal University, Nanjing, 210046, China; 2. Island Research Center, MNR, P.R.C, Fujian, 350400, China)

* Correspondence:
Qing Liu, Min Xu
E-mail: liuq@njnu.edu.cn; xumin0895@njnu.edu.cn

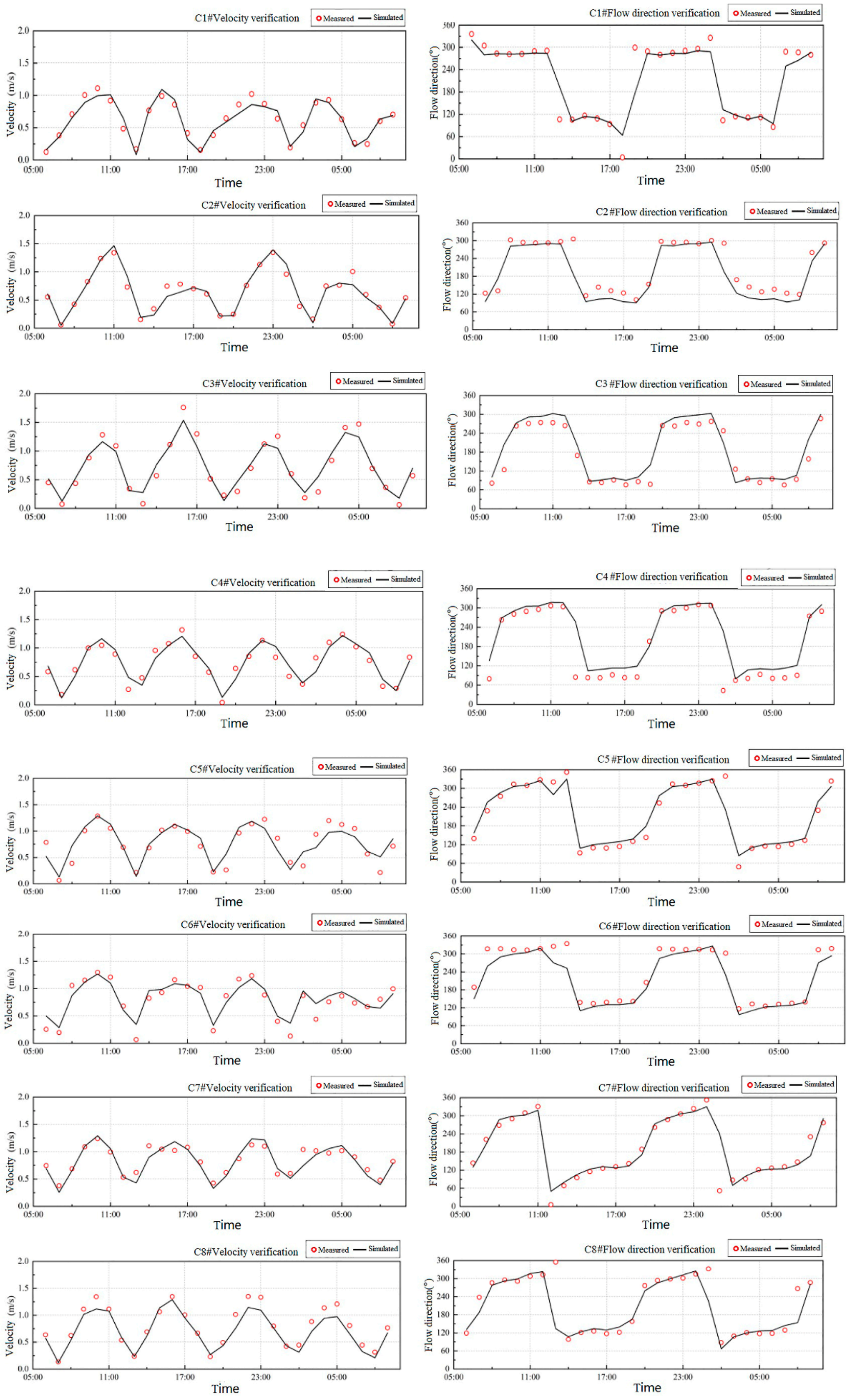

Figure S1. Validation of spring flow velocity and direction in April 2019.

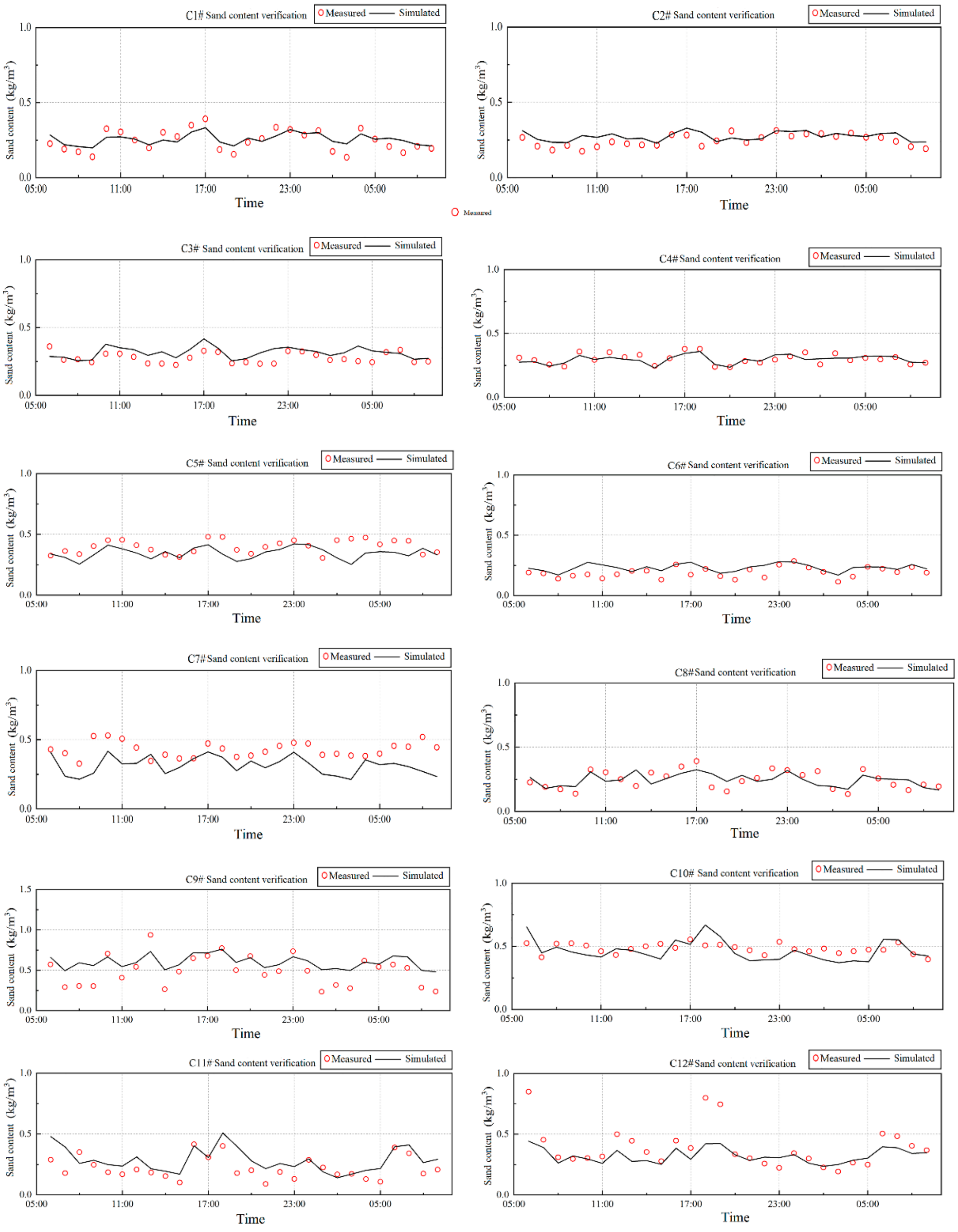

Figure S2. Validation of sediment concentration in water in April 2019

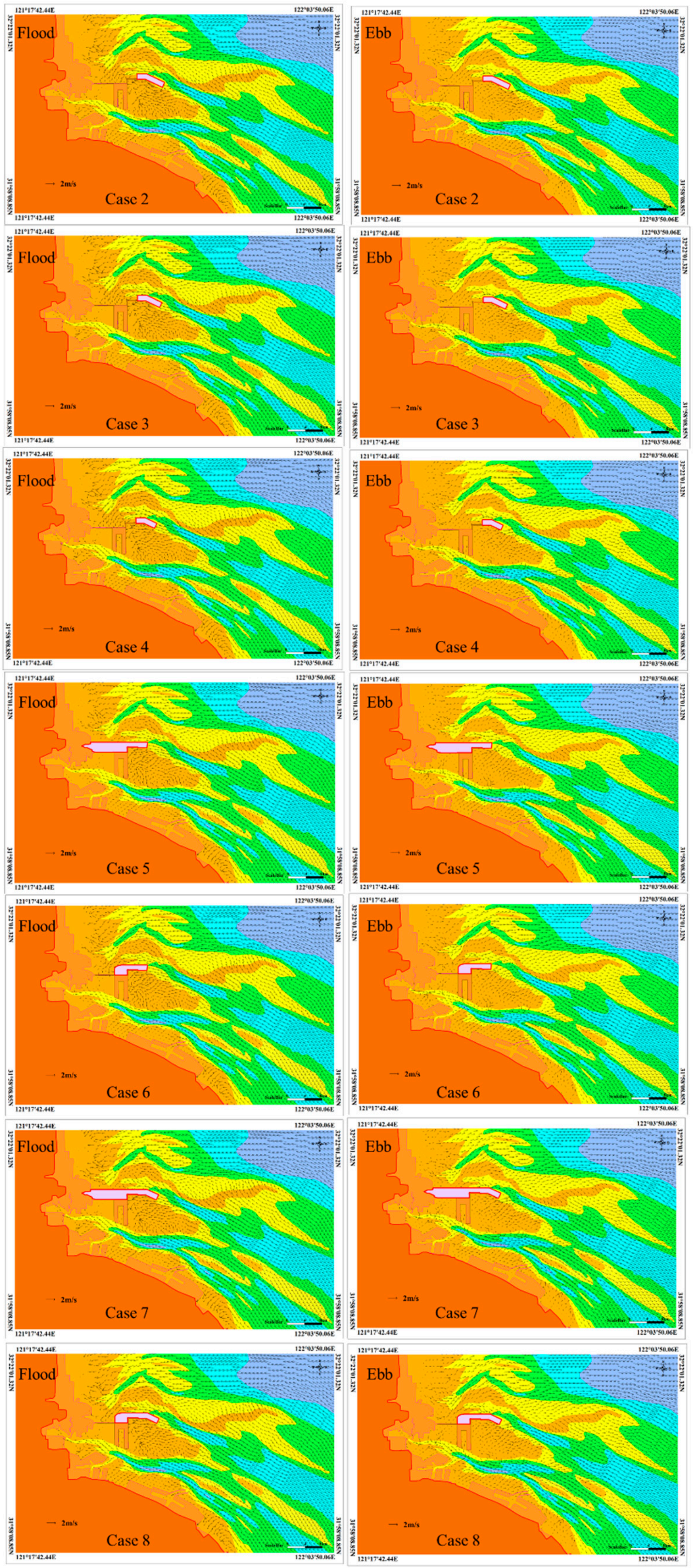

Figure S3. Influence of reclamation cases on the hydrodynamic environment

Table S1. Variation range of tidal flux at each section of the water channel

Case	time	Variation range of tidal flux at channel section (%)									
		DM1	DM2	DM3	DM4	DM5	DM6	DM7	DM8	DM9	DM10
Case 2	flood	**-10.56%**	-3.43%	-0.08%	-0.73%	-2.85%	-0.62%	-0.51%	-0.28%	2.67%	-1.49%
	ebb	**-11.58%**	-1.48%	-3.74%	-4.21%	-1.14%	-2.30%	-0.43%	-1.02%	3.20%	-0.64%
Case 3	flood	-7.03%	-2.78%	-0.04%	-0.77%	-2.43%	-0.63%	-0.43%	-0.26%	2.64%	-1.33%
	ebb	-8.26%	-1.31%	-3.62%	-3.85%	-1.03%	-1.89%	-0.34%	-0.91%	2.93%	-0.11%
Case 4	flood	-4.69%	-2.05%	0.31%	-0.64%	-1.95%	-0.51%	-0.32%	-0.23%	2.61%	-0.71%
	ebb	-6.13%	-1.05%	-3.88%	-3.44%	-0.78%	-1.52%	-0.29%	-0.78%	2.67%	0.39%
Case 5	flood	-4.90%	-2.52%	0.06%	-0.68%	-1.16%	-0.48%	-0.23%	-0.16%	**-7.41%**	-0.94%
	ebb	-7.37%	-1.58%	-3.39%	-2.59%	-0.60%	-1.04%	-0.31%	-0.80%	-3.55%	2.62%
Case 6	flood	-3.54%	-2.05%	0.09%	-0.63%	-1.02%	-0.43%	-0.23%	-0.14%	-0.97%	-0.79%
	ebb	-6.36%	-1.25%	-3.15%	-2.41%	-0.54%	-1.01%	-0.23%	-0.57%	2.56%	1.86%
Case 7	flood	-6.55%	-3.09%	0.23%	-0.84%	-2.36%	-0.70%	-0.38%	-0.29%	-1.19%	-1.15%
	ebb	**-11.20%**	-1.78%	-5.12%	-3.80%	-1.01%	-1.56%	-0.26%	-0.93%	2.42%	1.10%
Case 8	flood	-6.47%	-3.06%	0.24%	-0.84%	-2.35%	-0.70%	-0.38%	-0.29%	-0.83%	-1.15%
	ebb	-9.36%	-1.75%	-1.49%	-1.27%	-1.00%	-1.56%	-0.27%	-0.93%	2.54%	1.10%

Table S2. The variation of flood tide velocity in each case

Feature points	Case 2	Case 3	Case 3	Case 3	Case 3	Case 3	Case 3
1	**31.10%**	**34.92%**	**41.80%**	2.32%	4.20%	**37.52%**	**37.70%**
2	1.40%	-0.95%	-1.59%	-4.17%	-3.06%	-3.09%	-3.03%
3	-1.26%	-1.35%	-1.73%	-3.14%	-2.49%	-2.70%	-2.69%
4	-0.38%	-0.50%	-0.97%	-2.49%	-1.91%	-1.64%	-1.64%
5	-0.08%	-0.12%	-0.36%	-1.05%	-0.75%	-0.63%	-0.64%
6	-0.04%	-0.10%	-0.37%	-1.10%	-0.78%	-0.66%	-0.66%
7	0.00%	0.01%	-0.05%	-0.48%	-0.26%	-0.25%	-0.24%
8	0.03%	0.03%	-0.06%	-0.56%	-0.32%	-0.28%	-0.27%
9	0.07%	0.05%	-0.01%	-0.30%	-0.19%	-0.16%	-0.15%
10	0.04%	0.01%	-0.05%	-0.27%	-0.20%	-0.16%	-0.16%
11	-0.07%	-0.09%	-0.11%	-0.19%	-0.16%	-0.17%	-0.16%
12	-1.39%	-0.78%	0.09%	2.67%	1.85%	0.86%	0.81%
13	0.36%	0.31%	0.18%	0.45%	0.11%	0.33%	0.31%
14	1.03%	0.49%	-0.36%	-1.14%	-1.15%	-0.63%	-0.63%
15	1.71%	0.56%	-0.88%	-2.62%	-2.23%	-1.48%	-1.47%
16	1.65%	0.83%	-0.67%	-2.69%	-2.16%	-1.21%	-1.20%
17	2.15%	1.08%	-0.74%	-3.25%	-2.58%	-1.41%	-1.39%

18	0.51%	0.35%	-0.11%	-1.16%	-0.76%	-0.41%	-0.40%
19	0.44%	0.32%	-0.04%	-0.98%	-0.61%	-0.33%	-0.30%
20	0.26%	0.24%	0.05%	-0.70%	-0.38%	-0.23%	-0.21%
21	0.18%	0.15%	-0.02%	-0.67%	-0.38%	-0.28%	-0.26%
22	0.26%	0.21%	0.02%	-0.72%	-0.39%	-0.27%	-0.24%
23	-0.03%	-0.06%	-0.11%	-0.35%	-0.25%	-0.27%	-0.26%
24	-0.01%	-0.06%	-0.12%	-0.35%	-0.25%	-0.28%	-0.27%
25	0.07%	0.00%	-0.11%	-0.35%	-0.25%	-0.26%	-0.25%
26	-0.07%	-0.10%	-0.12%	-0.24%	-0.19%	-0.22%	-0.21%
27	-0.08%	-0.11%	-0.13%	-0.22%	-0.18%	-0.23%	-0.22%
28	-0.05%	-0.10%	-0.13%	-0.22%	-0.17%	-0.23%	-0.23%

Table S3. Tidal flux variation of each section under stable topography with fixed bed and equilibrium in case 8 (%)

Case 8	DM1		DM2		DM3		DM4		DM5	
	flood	ebb	flood	ebb	flood	ebb	flood	ebb	flood	ebb
Bed fixed	-6.47%	-9.36%	-3.06%	-1.75%	0.24%	-1.49%	-0.84%	-1.27%	-2.35%	-1.00%
equilibrium	-4.66%	-3.37%	-1.48%	-0.58%	1.69%	-2.06%	-0.51%	-2.76%	-1.41%	-0.73%
Case 8	DM6		DM7		DM8		DM9		DM10	
	flood	ebb	flood	ebb	flood	ebb	flood	ebb	flood	ebb
Bed fixed	-0.70%	-1.56%	-0.38%	-0.27%	-0.29%	-0.93%	-0.83%	2.54%	-1.15%	1.10%
equilibrium	-0.40%	-0.65%	-0.07%	-0.27%	-0.30%	-0.42%	-2.80%	-0.85%	-0.91%	-0.28%

Table S4. Characteristic point changes of flow velocity

**Case 8**	**1**		**2**		**3**		**4**		**5**		**6**	
	flood	ebb	flood	ebb	flood	ebb	flood	ebb	flood	ebb	flood	ebb
Bed fixed	37.70%	-10.23%	-3.03%	-7.46%	-2.69%	-6.45%	-1.64%	-3.20%	-0.64%	-2.01%	-0.66%	-1.66%
equilibrium	39.45%	4.70%	0.71%	-8.02%	-1.67%	-7.10%	-1.71%	-6.46%	-0.56%	-1.92%	-0.62%	-1.49%
**Case 8**	**7**		**8**		**9**		**10**		**11**		**12**	
	flood	ebb	flood	ebb	flood	ebb	flood	ebb	flood	ebb	flood	ebb
Bed fixed	-0.24%	-1.28%	-0.27%	-1.23%	-0.15%	-0.55%	-0.16%	-0.32%	-0.16%	-0.15%	0.81%	-3.14%

equilibrium	-0.53%	-1.30%	-0.60%	-1.24%	-0.36%	-0.53%	-0.34%	-0.23%	-0.34%	0.00%	0.58%	-1.59%
**Case 8**	**13**		**14**		**15**		**16**		**17**		**18**	
	flood	ebb	flood	ebb	flood	ebb	flood	ebb	flood	ebb	flood	ebb
Bed fixed	0.31%	-2.94%	-0.63%	-2.22%	-1.47%	-1.56%	-1.20%	-0.59%	-1.39%	-1.08%	-0.40%	-1.50%
equilibrium	1.89%	-2.38%	0.07%	-3.11%	0.21%	-0.09%	-0.37%	-1.13%	-0.65%	0.03%	-0.79%	-0.53%
**Case 8**	**19**		**20**		**21**		**22**		**23**		**24**	
	flood	ebb	flood	ebb	flood	ebb	flood	ebb	flood	ebb	flood	ebb
Bed fixed	-0.30%	-1.05%	-0.21%	-1.28%	-0.26%	-1.06%	-0.24%	-0.99%	-0.26%	-0.23%	-0.27%	-0.05%
equilibrium	-1.00%	-0.59%	-0.79%	-1.10%	-0.93%	-0.92%	-1.09%	-0.86%	-0.60%	-0.18%	-0.69%	0.03%
**Case 8**	**25**		**26**		**27**		**28**					
	flood	ebb	flood	ebb	flood	ebb	flood	ebb				
Bed fixed	-0.25%	0.31%	-0.21%	0.03%	-0.22%	0.21%	-0.23%	0.42%				
equilibrium	-0.77%	0.35%	-0.47%	0.17%	-0.50%	0.33%	-0.58%	0.54%				
